# Phase 1 studies of the indenoisoquinolines LMP776 and LMP744 in patients with solid tumors and lymphomas

**DOI:** 10.1007/s00280-025-04778-5

**Published:** 2025-05-29

**Authors:** Geraldine O’Sullivan Coyne, Shivaani Kummar, Larry V. Rubinstein, Deborah Wilsker, Nancy Moore, Murielle Hogu, Richard Piekarz, Joe Covey, Jan H. Beumer, Katherine V. Ferry-Galow, Liza C. Villaruz, Melinda G. Hollingshead, Julianne L. Holleran, Joshua J. Deppas, Yves Pommier, Brian Ko, Barry C. Johnson, Ralph E. Parchhment, Percy Ivy, James H. Doroshow, Alice P. Chen

**Affiliations:** 1 Division of Cancer Treatment and Diagnosis, National Cancer Institute, National Institutes of Health, 31 Center Drive, Building 31, Room 3A44, Bethesda, MD 20892 USA; 2 Biometric Research Program, NCI, NIH, Bethesda, MD USA; 3 Clinical Pharmacodynamics Biomarker Program, Applied/Developmental Research Directorate, Frederick National Laboratory for Cancer Research, Frederick, MD USA; 4 Cancer Therapeutics Evaluation Program, NCI, NIH, Bethesda, MD USA; 5 UPMC Hillman Cancer Center, Pittsburgh, PA USA; 6 Department of Pharmaceutical Sciences, University of Pittsburgh School of Pharmacy, Pittsburgh, PA USA; 7 Division of Hematology-Oncology, Department of Medicine, University of Pittsburgh School of Medicine, Pittsburgh, PA USA; 8 Biological Testing Branch, NCI, NIH, Frederick, MD USA; 9 Developmental Therapeutics Branch and Laboratory of Molecular Pharmacology, Center for Cancer Research, NCI, NIH, Bethesda, MD USA; 10 Center for Cancer Research, NCI, NIH, Bethesda, MD USA; 11 Present Address: Knight Cancer Institute, Oregon Health and Science University, Portland, OR USA

**Keywords:** Topoisomerase, Indenoisoquinoline, Pharmacodynamic, Clinical trial

## Abstract

**Purpose:**

Indenoisoquinolines are a class of topoisomerase I (TOP1) inhibitors designed to overcome clinical limitations of camptothecins. Three indenoisoquinolines (LMP400, LMP776, and LMP744) demonstrated activity in murine models and a comparative canine lymphoma study. Clinical data for LMP400 were previously reported (NCT01051635). The maximum tolerated dose (MTD), safety, and clinical data from phase 1 studies of LMP776 (NCT01051635) and LMP744 (NCT03030417) are reported herein.

**Methods:**

Patients ≥ 18 years of age with advanced, refractory solid tumors or lymphomas received either LMP776 (n = 34) or LMP744 (n = 35) intravenously following a Simon accelerated titration design. Both LMP776 and LMP744 were administered daily for 5 days (QDx5) in 28-day cycles. Adverse events and clinical responses were evaluated according to CTCAE and RECIST v1.1 criteria, respectively. Pharmacokinetic and pharmacodynamic changes were evaluated.

**Results:**

The MTD of LMP776 was 12 mg/m^2^/day and that of LMP744 was 190 mg/m^2^/day. Dose-limiting toxicities (DLTs) for LMP776 included hypercalcemia, anemia, and hyponatremia; DLTs for LMP744 included hypokalemia, anemia, and weight loss. There was 1 confirmed partial response (cPR) among 35 patients receiving LMP744 (overall response rate 3%) and no objective responses in patients receiving LMP776. Tumor biopsies from the patient with cPR demonstrated high baseline expression of SLFN11 and a unique pattern of pharmacodynamic responses, including increased RAD51, phosphorylated KAP1 (pKAP1), γH2AX, and cleaved caspase-3 (cCasp3).

**Conclusion:**

MTDs and safety profiles are reported for LMP776 and LMP744. Target engagement by an indenoisoquinoline was measured for the first time in human samples.

**Supplementary Information:**

The online version contains supplementary material available at 10.1007/s00280-025-04778-5.

## Introduction

DNA topoisomerase I (TOP1) relaxes DNA supercoiling by inducing single-strand breaks via nucleophilic attack on the DNA backbone, forming a covalently linked cleavage complex (TOP1cc) between TOP1 and the DNA [1]. The chemotherapeutic drugs camptothecin (CPT) and its derivatives topotecan and irinotecan are TOP1 inhibitors that intercalate into DNA at the cleavage site and inhibit the reverse reaction in which the cleaved DNA strand is re-ligated and TOP1 released [2–4]. Trapped TOP1cc’s block DNA replication and transcription and lead to apoptosis (Supplementary Figure S1) [5]. Clinical use of CPT derivatives is limited by several factors, including (1) instability of the α-hydroxylactone E-ring leading to inactivation of the compounds [6, 7], (2) rapid dissociation of the drugs from the TOP1cc’s [3, 7], and (3) export of the drugs from cells by membrane pumps [8]. Also, topotecan and irinotecan treatment are both associated with hematologic toxicities, and irinotecan treatment is associated with diarrhea; both toxicities can be severe enough to require dose reduction, treatment interruption, or discontinuation [9].

Indenoisoquinolines (IIQs) are a class of TOP1 poisons designed to have more favorable pharmacologic characteristics than their CPT-derived predecessors [10]. Notably, IIQs are more chemically stable than CPT, topotecan, or irinotecan due to the replacement of the α-hydroxylactone E-ring moiety [6]. IIQs demonstrated DNA cleavage patterns that differ from CPT and its derivatives [11]. The TOP1cc’s trapped by IIQs persist longer than those trapped by CPT, topotecan, or irinotecan in cells [12]. These favorable characteristics spurred a drug discovery effort that produced over 700 IIQ analogues [13]. In vitro activities of many of these compounds were evaluated in the NCI-60 cell line panel and LMP400 (indotecan, NSC 743400), LM776 (indimitecan, NSC 725776), and LMP744 (NSC 706744) were selected for further preclinical development based on favorable activity and pharmacologic properties; these preclinical studies in mouse models demonstrated sufficient target engagement and anti-tumor efficacy to initiate clinical trials of LMP400 and LMP776 (NCT01051635) [11, 12, 14, 15]. In conjunction with the human clinical trial, the efficacies of these compounds and LMP744 were evaluated in client-owned canines (i.e., companion animals) with naturally occurring lymphomas through the NCI Comparative Oncology Program [16]. Given the promising response rate of LMP744 (68.4%, 13 of 19) among canines with lymphomas compared to LMP400 or LMP776 (33.3% [9 of 27]; and 29.2% [7 of 24] for LMP400 and LMP776, respectively) [16], a clinical trial of LMP744 was initiated subsequent to the trial evaluating LMP400 and LMP776.

Here, we present safety and clinical data for first-in-human clinical trials of LMP776 (NCT01051635) and LMP744 (NCT03030417) alongside previously published LMP400 data [17]. We also present primary, secondary, and tertiary pharmacodynamic effects in the nuclei of tumor cells induced by LMP744 treatment. Target engagement by LMP744 was assessed directly by measuring nuclear levels of TOP1 and TOP1cc’s at single-strand DNA breaks. Collision of a replication fork with trapped complexes causes double-stranded breaks (DSBs) [18] that initiate a pharmacodynamic cascade assessed by measuring nuclear levels of DSB repair markers (γH2AX, pNBS1, RAD51 [19]) as well as pSer824-KAP1, a downstream target of ATM. Phosphorylated histone γH2AX accumulates near double-stranded DNA breaks, functions in signal amplification, and serves as a scaffold for other DNA repair factors; it has long been used as a marker of double-stranded DNA breaks [15, 20–22]. KRAB-associated protein 1 (KAP1) relaxes chromatin to facilitate DNA repair [23, 24]. NBS1 forms a complex with MRE11 and RAD50 that can repair DSBs via homologous recombination [25]. RAD51 also catalyzes DSB repair via homologous recombination through interaction with BRCA2 [26, 27]. SLFN11 blocks DNA repair pathways and can force DNA replication to continue despite damage, leading to further damage and instability [28]. Lethal levels of DSBs ultimately lead to apoptosis, which was assessed by reflex testing of γH2AX-positive tumors for co-localized cCasp3. cCasp3 has several functions in initiating and maintaining apoptosis and the presence of cCasp3 blebbing is a validated marker of apoptosis [29].

## Patients and methods

### Trial design and objectives

The primary objectives of each study included establishing safety, tolerability, MTD, and recommended phase 2 dose (RP2D) of LMP776 and LMP744 as single agents. The LMP744 study included evaluation of tumor pharmacodynamics as an exploratory objective. Both trials were opened at the National Cancer Institute’s (NCI) Developmental Therapeutics Clinic, and the LMP744 trial was also opened at the University of Pittsburgh Hillman Cancer Center. Both trials used an accelerated dose escalation designs (Simon design 4 for LMP776, Simon design 3 for LMP744) to determine the MTD [30]. Dose levels are listed in Supplementary Table 1. Once the MTD was determined, additional patients were enrolled at that DL in an expansion cohort to evaluate pharmacodynamic changes as described below. LMP776 and LMP744 were administered intravenously through a central line via a 1-h infusion on days 1–5 (QDx5) in 28-day cycles. Both agents were supplied by the NCI Division of Cancer Treatment and Diagnosis (DCTD). The studies were approved by the NIH Institutional Review Board and conducted in accordance with the Declaration of Helsinki.

### Eligibility criteria

Patients were enrolled in either NCT01051635 evaluating LMP776 or NCT03030417 evaluating LMP744; these trials were conducted sequentially. Both trials required that patients be ≥ 18 years of age with solid tumors or lymphomas refractory to other treatments. Patients were required to have adequate performance status (Eastern Cooperative Oncology Group [ECOG] performance status ≤ 2), bone marrow function, and organ function as described in the Supplementary Methods. Patients in expansion phases at the MTD were required to have disease amenable to biopsy collection and be willing to undergo research biopsies. All patients provided written informed consent.

### Clinical evaluations

Tumor responses were evaluated by serial CT scans according to RECIST v1.1 criteria [31]. CT scans were performed every other cycle during the first year, then every 3 cycles after the first year. Toxicities associated with LMP776 were evaluated according to criteria specified in CTCAE version 4.0; toxicities associated with LMP744 were evaluated according to the criteria specified in CTCAE version 4.03 (on or before March 31, 2018) or version 5.0 (on or after April 1, 2018). Criteria for all versions can be found at https://ctep.cancer.gov/protocolDevelopment/electronic_applications/ctc.htm. Any patient who received a dose of either agent was considered eligible for toxicity evaluation.

### Pharmacokinetics

Blood was collected during cycle 1 in the LMP744 study on C1D1 at the end of infusion (EOI), then again at 0.25, 0.5, 1, 2, 4, and 6 h after EOI. Blood was also collected prior to infusion and at EOI on days 2–5 during cycle 1, then again at 1 week (168 h) after C1D1 EOI. Samples were collected in EDTA tubes with 2 mL of blood per sample. Samples were centrifuged and plasma was stored at − 70 °C. Samples were analyzed using a validated LC–MS or LC–MS/MS method for each IIQ as previously described [32]. Plasma-time data were analyzed using WinNonlin (Pharsight, Mountain View, CA). AUC values were calculated by non-compartmental analysis and end-of-infusion concentrations were measured on days 1 and 5 of cycle 1. Sample collection and analysis of LMP776 pharmacokinetics were performed as previously described [17].

### Antibody validation for pharmacodynamic biomarker assays

Several PD biomarker assays for DNA damage response and apoptosis, along with their key assay reagents, have already been analytically validated and proven fit-for-purpose for evaluating human tumor specimens from clinical core needle biopsies: pNBS1, RAD51, and γH2Ax (with or without co-localized cCasp3) [15–17, 19, 29, 32]. Additional pharmacodynamic biomarker assays for DNA damage response were used clinically for the first time in the IIQ trials reported herein: nuclear SLFN11 with monoclonal antibody clone D8W1B; nuclear TOP1 and its cleavable complex (TOP1cc) with monoclonal antibody clones EPR5376-(2) and 1.1A, respectively; and nuclear pKAP1 with monoclonal antibody clone EPR5248. These monoclonal antibodies were selected from among commercial candidates based on performance during analytical validation as assay key reagents and then performance in quantitative immunofluorescence (qIF) microscopy (in immunohistochemistry for anti-SLFN11). Antibody specificity and sensitivity were established by proper reactivity with positive and negative control cell lines and xenograft tissues when compared to isotype control antibodies, not only in the qIF platform with secondary antibody conjugates as reporters, but also with Western blotting using conventional methods.

### Pharmacodynamic biomarker analyses

The LMP744 protocol collected pre-treatment (baseline) and on-treatment paired biopsies from patients in the expansion cohort. Core needle tumor biopsies (18-gauge) were collected and flash-frozen at point of collection per NCI SOPs (https://dctd.cancer.gov/ResearchResources/biomarkers/docs/par/SOP340507_Biopsy_Frozen.pdf) as described to preserve labile post-translational modifications such as phosphorylation [19, 33]. Biopsy pairs were thawed in formalin, fixed in paraffin embedded together with control tissues following NCI SOP340550 (https://dctd.cancer.gov/ResearchResources/biomarkers/DDR3/SOP340550_Biopsy_Section_Testis_Jejunum_Controls.pdf). TOP1 and TOP1cc were measured with a cocktail containing custom-conjugated rabbit monoclonal antibody anti-TOP1-DIG (Abcam, EPR5376 (2)) and mouse monoclonal antibody anti-TOP1cc (clone 1.1A [34, 35], EMD Millipore) that were detected with reporter secondary antibodies AlexaFluor-647 IgG fraction monoclonal mouse anti-digoxin (Jackson ImmunoResearch) and goat/anti-mouse AlexaFluor-488, respectively. Phosphorylated KAP1 (pKAP1, phosphorylated at S824) was measured using custom-conjugated pKAP1-pS824-DNP (Abcam, EPR5248) and detected with AlexaFluor-488 conjugated with anti-dinitrophenyl-KLH rabbit IgG Antibody Fraction (DNP-488, ThermoFisher). Validated immunofluorescence assays (IFAs) were used to measure levels of γH2AX, phosphorylated NBS1 (pNBS1, phosphorylated at Ser343), and RAD51 [15, 17, 19, 29]. Antibodies in all clinical immunofluorescence staining protocols were used as a cocktail with β-catenin as a tumor marker as previously described [36]. After staining with primary antibodies, washing, and staining with secondary antibodies, slides were rinsed in 1X PBS and blotted to remove excess liquid. Slides were cured overnight with Prolong Gold Antifade Reagent (Invitrogen) in the dark and imaged the following day. Confocal images (12-bit) were acquired at 20X using a Nikon A1 scan head on a Nikon 90i microscope at 0.24 µm/pixel to measure nuclear TOP1 and nuclear foci of TOP1cc and RAD51. Fluorescent images were acquired at 20X using an Axioscan7 (Zeiss) to measure nuclear content of pKAP1, γH2AX, and pNBS1. TOP1, γH2AX, pNBS1, and pKAP1 were measured as the percentage of DAPI-stained tumor cell nuclear area that was positive for the biomarker (% NAP) [19]; RAD51 [19] and TOP1cc was measured as a function of the percentage of nuclei with ≥ 5 foci (RAD51) or ≥ 19 foci (TOP1cc). Apoptosis was measured using a validated IFA of tumor cells positive for both cCasp3 blebbing in the cytoplasm and γH2AX in the nucleus [29]. Anti-SLFN11 rabbit monoclonal antibody (Cell Signaling Technology, clone D8W1B) was detected by immunohistochemistry (IHC) using Bond Polymer Detection kit (Leica) and scanned in 20X widefield Aperio (Leica), and the distribution of staining intensity of tumor cell nuclei graded as 0, 1 + , 2 + or 3 + was converted into an H-score. With 6 paired biopsies, there was 90% power to detect a treatment effect equivalent to 1.85 standard deviations (SDs) with the paired 2-sample t-test at the 1-sided 0.05 significance level. Comparisons of pre-treatment and on-treatment (C1D2) samples were performed using GraphPad Prism version 9.3.1.

### Preclinical models

Patient derived xenograft (PDX) models were implanted into gender-matched NSG mice (NOD.Cg-Prkdc^scid^Il2rg^tm1Wjl^/SzJ; NCI Animal Production Program, Frederick, MD) as serially passaged tumor fragments using cryopreserved fragments as needed to avoid excessive in vivo passaging (10 passages maximum) as described in the PDMR standard operating procedures (https://pdmr.cancer.gov/sops). All mice were generated from in-house breeding colonies. The NSG mice were maintained as inbred colonies. Mice were housed in sterile individually ventilated polycarbonate cages on RAIR HD SuperMouse 750™ ventilated racks outfitted with automatic watering and HEPA-filtered supply and exhaust air (Lab Products, Aberdeen, MD). All animals were maintained in a strict barrier facility on a 12-h light/dark cycle and were provided with sterilized food *ad libitum*. LMP744 (NSC 706744) and olaparib (NSC 753686) were obtained through the NCI Developmental Therapeutics Program (DCTD, NCI; Rockville, MD). Models, dosing regimens, and number of mice in each experiment are provided in Supplementary Table 2. Mice bearing 144,126–210-T tumors were staged to a median tumor size of 200 mm^3^, at which time the animals were randomized into four treatment groups using a commercial software program (Study Director, Studylog Systems, Inc.) and treatment began. Tumor size was monitored over time by pre-scheduled bidirectional caliper measurements, and the tumor volumes (mm^3^) were calculated as (tumor length in mm × [tumor width in mm]^2^)/2 [37]. The Frederick National Laboratory for Cancer Research is accredited by the Association for Assessment and Accreditation of Laboratory Animal Care International and follows the USPHS Policy for the Care and Use of Laboratory Animals. All the studies were conducted according to an approved animal care and use committee protocol in accordance with the procedures outlined in the ‘‘Guide for Care and Use of Laboratory Animals’’ [38].

## Results

### Patients

Thirty-four patients enrolled on the LMP776 trial between February 2010 and September 2016 at the National Cancer Institute (NCI). The median age at enrollment was 56.5 years (range 22–72 years). A total of 36 patients enrolled on the LMP744 trial at the NCI or the University of Pittsburgh Hillman Cancer Center between February 2017 and June 2022; one patient enrolled but did not start treatment and was not included in the analyses. The median age of these patients was 61 years (range 35–81 years). Demographic data for patients in both trials are provided in Table 1. Fifteen of 34 patients in the LMP776 trial and 26 of 36 patients in the LMP744 trial had received prior TOP1 inhibitor therapy (e.g., topotecan or irinotecan). Colon (n = 6) and pancreas (n = 3) were the most prevalent primary tumor sites in the LMP776 study, while colon (n = 11), colorectal (n = 5), and lung (n = 4) were the most prevalent primary tumor sites in the LMP744 study (Supplementary Table S3). A patient with cutaneous T-cell lymphoma was treated with LMP776, and a patient with Hodgkin lymphoma was treated with LMP744.

**Table 1:** Patient demographics

	LMP776	LMP744
Number of patients enrolled	34	36
Female / Male	12 / 22	9/27
Median age, years (range)	56.5 (22–72)	61 (35–81)
Race		
White	21	29
Black or African-American	9	6
Asian	2	1
Unknown	2	–
Ethnicity		
Not Hispanic or Latinx	30	32
Hispanic or Latinx	4	4
Prior TOP1 inhibitor, n (%)	15 (44)	26 (72)
Irinotecan combination	13^a^ (38)	19^c^ (53)
Irinotecan monotherapy	0 (0)	4 (11)
Topotecan combination	3^b^ (9)	2^d^ (6)
Topotecan monotherapy	1 (3)	0 (0)
Unspecified TOP1 inhibitor	0 (0)	1 (3)

^a^Includes modified FOLFIRI (n = 9), FOLFIRI (n = 4), irinotecan + cetuximab (n = 2), FOLFIRINOX (n = 1), irinotecan + bevacizumab (n = 1), irinotecan + carboplatin (n = 1), and irinotecan + 5-fluorouracil + bevacizumab (n = 1); the sum of these numbers is greater than the 13 patients listed in the table as patient 50 received 3 prior irinotecan regimens and patients 5, 14, 44, and 46 each received 2 prior irinotecan combination regimens

^b^Includes topotecan + veliparib (n = 3)

^c^Includes modified FOLFIRI (n = 9), FOLFIRI (n = 5), FOLFIRINOX (n = 2), modified FOLFOXIRI (n = 2), FOLFIRI (n = 1), irinotecan + fluorouracil (n = 1), and irinotecan + bevacizumab (n = 1); the sum of these numbers is greater than the 19 patients listed in the table as patients 25 and 34 each received 2 prior irinotecan combination regimens

^d^Includes topotecan + berzosertib (n = 1) and topotecan + bevacizumab (n = 1)

### MTD determination and adverse events

The MTD of LMP776 was determined to be 12 mg/m^2^/day IV on a QDx5 schedule (DL7). Dose escalation reached DL8 (16 mg/m^2^/day IV). Hypercalcemia and hyponatremia were dose-limiting. Grade 3 non-hematologic toxicities at DL8 included hypokalemia, hyponatremia, and hypophosphatemia; these AEs occurred at grade 3 in one patient each (Table 2, Supplementary Table S4). Grade ≥ 3 electrolyte AEs were not considered dose-limiting if they were corrected to grade 1 or baseline within 24 h. The most prevalent grade ≥ 2 AEs across all DLs were anemia (50%), lymphopenia (38%), and thrombocytopenia (18%). One patient receiving LMP776 enrolled at DL8 required a dose reduction.

**Table 2:** Most prevalent grade ≥ 2 adverse events (≥ 10% of patients, highest grade per patient)

Adverse event	Total (%)	Grade	DL1-DL2 (n = 8)	DL3-DL4 (n = 6)	DL5-DL6 (n = 6)	DL7 (n = 7)	DL8 (n = 7)
*LMP776*							
Anemia	17 (50)	2	5	2	3	1	
		3		1		1	4
Lymphopenia	13 (38)	2	2	1			
		3		2	1	2	3
		4					2
Thrombocytopenia	6 (18)	2					1
		3				1	3
		4					1
Neutropenia	5 (15)	2			1		1
		3					2
		4					1
Hypophosphatemia	4 (12)	2	1			1	1
		3					1

Adverse event	Total (%)	Grade	DL1-DL5 (n = 7)	DL6 (n = 18)	DL7 (n = 11)
*LMP744*					
Lymphopenia	14 (39)	2	1	1	4
		3		5	2
		4		1	
Anemia	13 (36)	2	2	4	7
Fatigue	8 (22)	2		6	2
GGT increased	6 (17)	2	1	3	
		3		1	1
Nausea	6 (17)	2		3	1
		3		1	1
Hypophosphatemia	6 (17)	2	1	4	1
Vomiting	6 (17)	2	1	4	1
Creatinine increased	5 (14)	2		3	2
Neutropenia	5 (14)	2		3	
		3		1	
		4		1	
Leukopenia	5 (14)	2		3	1
		4		1	
Dehydration	4 (11)	2		1	1
		3		1	1

The MTD of LMP744 was determined to be 190 mg/m^2^/day (DL6) IV on a QDx5 schedule. Dose escalation reached 260 mg/m^2^/day IV (DL7). Grade 3 non-hematologic DLTs at DL7 included elevated ALT, AST, and GGT in one patient; dose-limiting grade 3 nausea, vomiting, and dehydration occurred in another patient who was hospitalized with these symptoms and died of a pulmonary embolism; the death was considered possibly related to treatment (Table 2, Supplementary Table S5). The most frequently occurring grade ≥ 2 AEs across all DLs were lymphopenia (39%), anemia (36%), and fatigue (22%). Nausea was more prevalent in patients receiving LMP744 (17%, any grade) than in patients receiving LMP400 (5%) or LMP776 (6%, Supplementary Table S6). Notably different from irinotecan, only 1 patient in each study experienced diarrhea (both at grade 2). Eleven patients receiving LMP744 required dose reductions; all dose reductions occurred in patients enrolled at either DL6 (n = 6) or DL7 (n = 5).

### Clinical outcomes

**Fig. 1:**
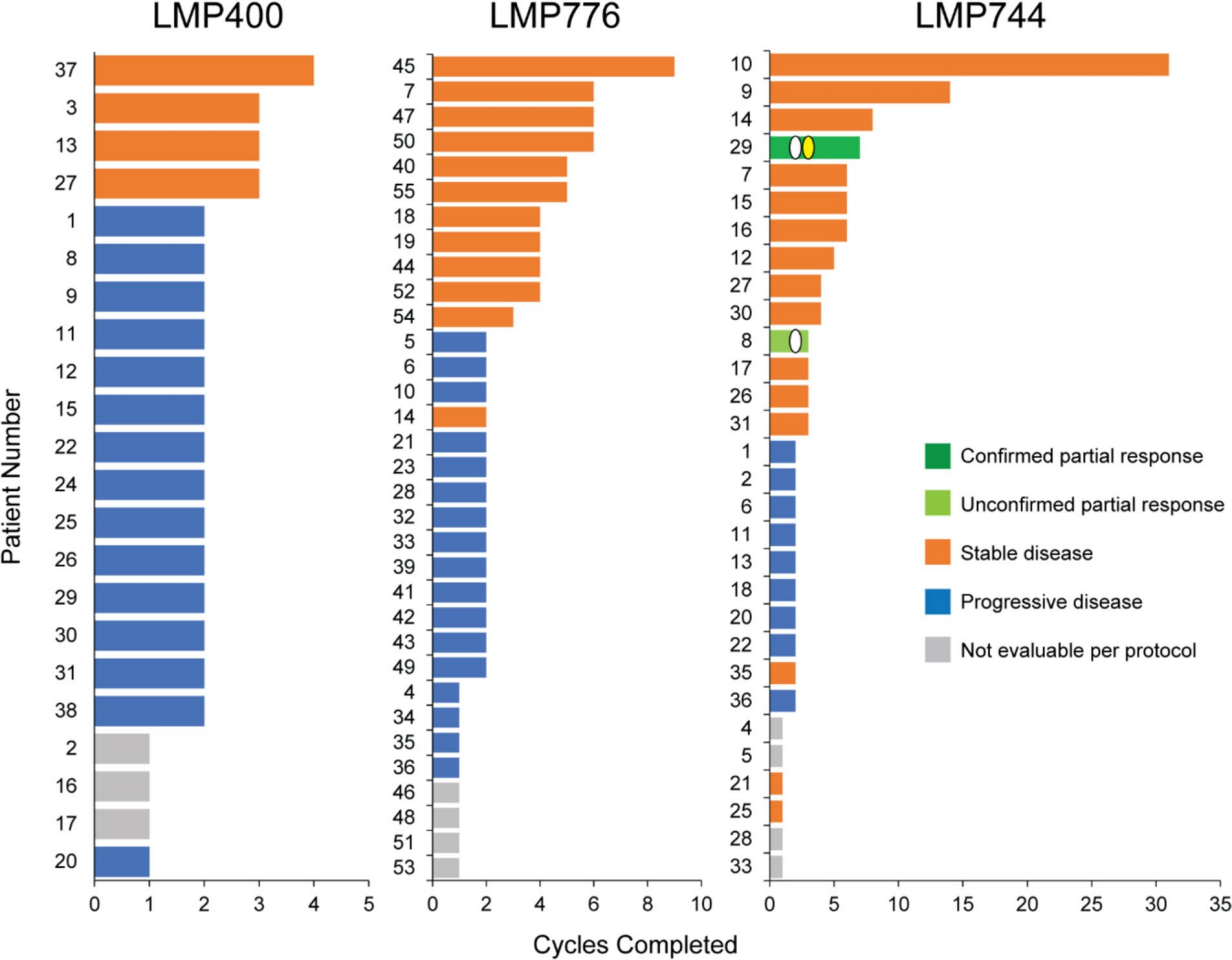
Cycles of treatment completed for each patient. Bars are color-coded based on best responses as indicated for LMP400 (left panel, [17]), LMP776 (middle panel), or LMP744 (right panel). White ovals indicate the times of initial partial response, the yellow oval indicates the time of confirmed partial response

For both LMP776 and LMP744, patients completed a median of 2 cycles of treatment (ranges 1–9 cycles for LMP776 and 1–31 cycles for LMP744; Fig. 1, with previously published LMP400 data for comparison [17]). On the LMP776 study, the longest duration of SD was 9 cycles (patient 45, pancreatic cancer). Although no objective responses were measured, 12 of 34 patients (35%) experienced stable disease. Two patients receiving LMP744 had a partial response, although only one was confirmed with a subsequent scan. Patient 29 (NSCLC with neuroendocrine features that converted to SCLC) achieved an initial PR prior to starting cycle 3 that was confirmed prior to starting cycle 4 (Fig. 1). This patient eventually came off study due to disease progression after completing 7 cycles. Patient 8 (Hodgkin lymphoma) also achieved an initial PR prior to starting cycle 3; however, this patient experienced adverse events that required removal from the study before a confirmatory CT scan could be performed. Patient 10 (low-grade appendiceal mucinous neoplasm) completed 31 cycles of treatment with SD. LMP744 was the only IIQ in the 3 LMP studies, including the 2 presented here and a study of alternative dosing regimens of LMP400 (NCT01794104), to result in an objective response and the longest duration of SD of the three IIQ compounds (Fig. 1). Among patients with PRs (confirmed or unconfirmed), patient 29 had received prior treatment with topotecan + veliparib while patient 8 received no prior TOP1 inhibitor. Of the 12 patients with stable disease for ≥ 3 cycles, 7 had received prior TOP1 inhibitor therapy (patients 7 [unspecified TOP1 inhibitor], 9 [FOLFIRI + bevacizumab], 14 [FOLFIRI], 16 [FOLFIRI + bevacizumab], 17 [FOLFIRI], 30 [irinotecan monotherapy], and 31 [topotecan + bevacizumab]),

### Pharmacokinetics

**Fig. 2:**
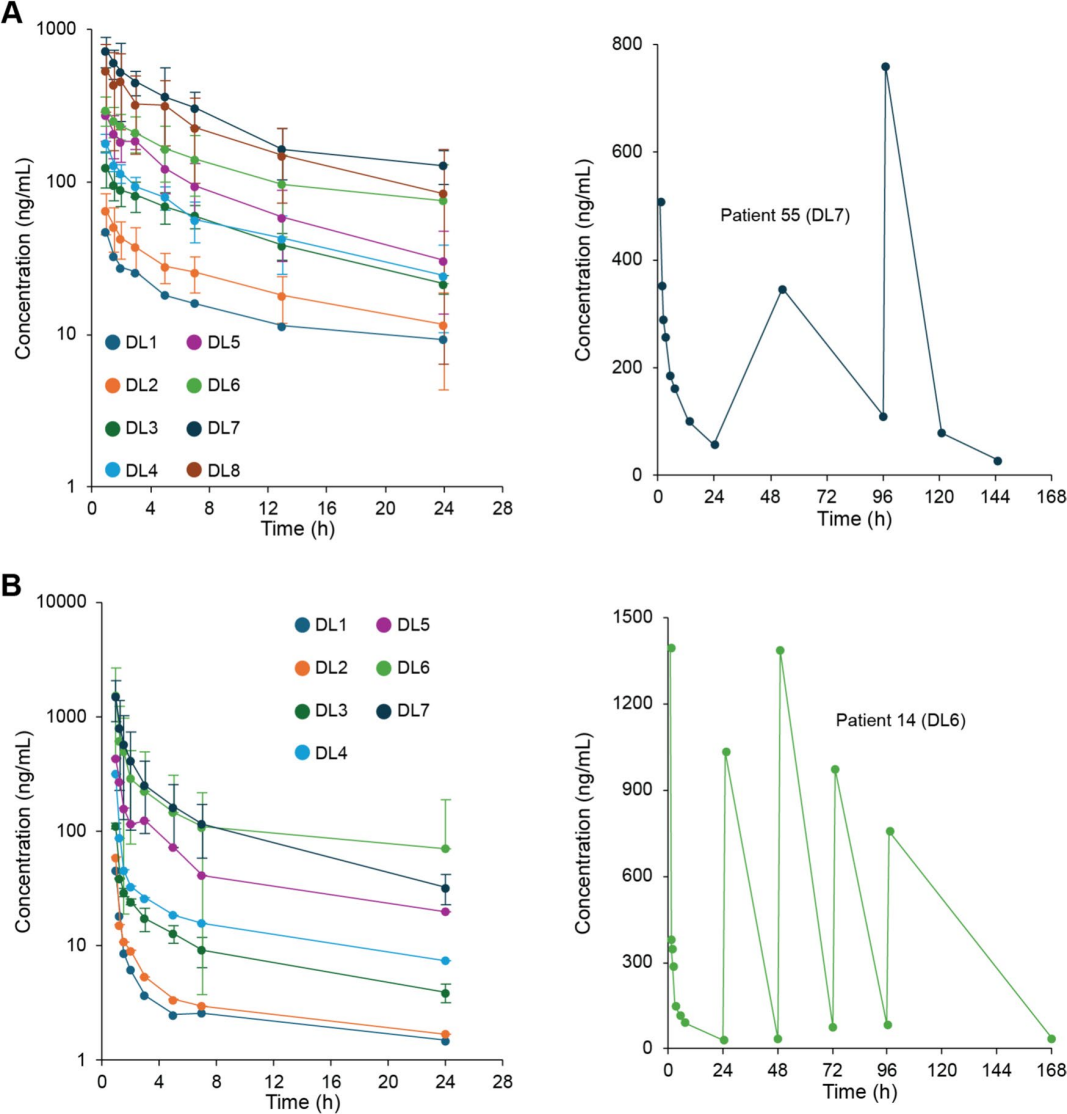
Pharmacokinetics of LMP776 and LMP744. **A** *left:* Plasma concentrations of LMP776 over the first 24 h after EOI on day 1 increase with dose level up to DL7; DL8 concentrations overlap with DL7 values. *right:* Representative concentration–time curve (patient 55) during cycle 1 at the MTD (DL7) of LMP776. **B** *left:* Plasma concentrations of LMP744 over the first 24 h after EOI on day 1 increase with dose level up to DL6; DL7 concentrations overlap with DL6 values. *right:* Representative concentration–time curve (patient 14) during cycle 1 at the MTD (DL6) of LMP744

Pharmacokinetic parameters were determined from concentration–time curves of blood levels in patients receiving either LMP776 or LMP744. Plasma concentrations of LMP776 increased with dose (Fig. 2A, Supplementary Figure S2). At the MTD (DL7, 12 mg/m^2^, n = 6), the mean half-life was 12.6 h (range 8.2–16.0 h, Supplementary Table S7) and half-life was independent of dose (Supplementary Table S7 and Figure S2). Plasma concentrations of LMP744 also increased with dose (Fig. 2B). The mean half-life of LMP744 at the MTD (DL6, 190 mg/m^2^, n = 16) was 13.6 h (range 7.2–23.4 h). Complete pharmacokinetic parameters are reported in Supplementary Table S7.

### Pharmacodynamics of indenoisoquinolines in tumor biopsies

**Fig. 3:**
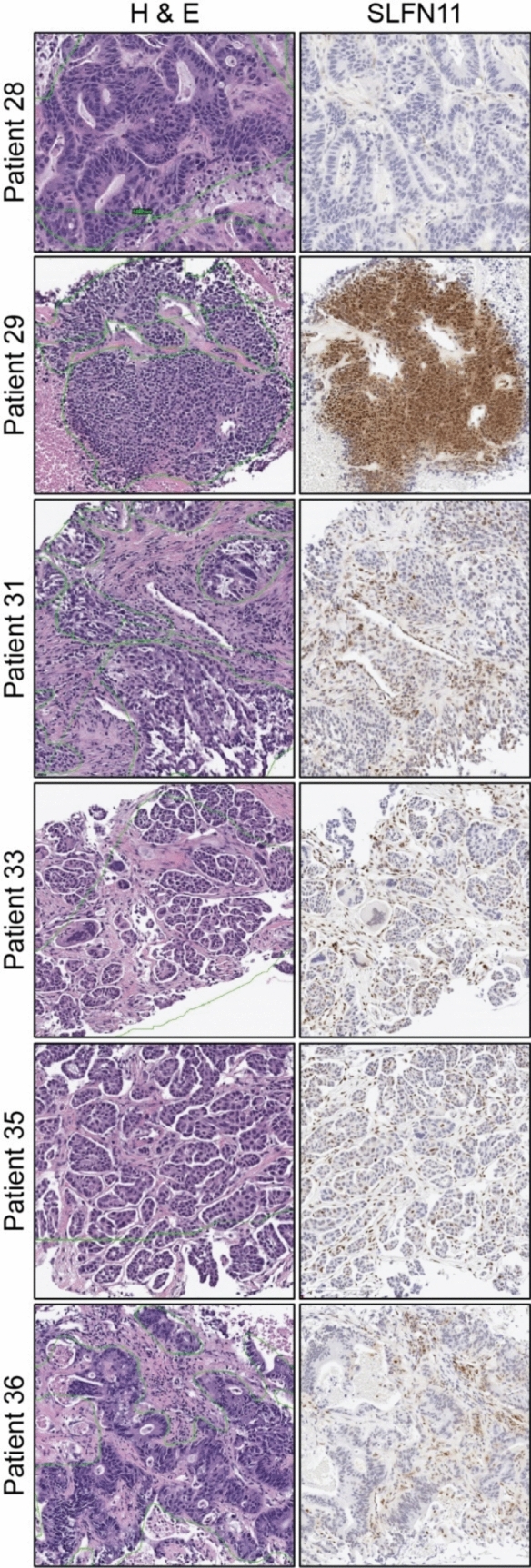
Immunohistochemical analysis of SLFN11 expression in baseline (pre-treatment) tumor biopsies. Sections of pre-treatment tumor biopsies from 6 patients in the expansion cohort of LMP744 at MTD (DL6; 190 mg/m^2^/day) were stained either with (*left*) hematoxylin and eosin or (*right*) anti-SLFN11 monoclonal antibody. Expression was low in patients with colorectal cancer (patients 28 and 36) and ovarian cancer (patients 31 and 33). Expression of SLFN11 in patients with ampullary cancer (patient 35) has not been reported. SLFN11 expression was high in the biopsy from patient 29, who exhibited the only cPR

Nuclear SLFN11 is a predictive biomarker for response to topoisomerase inhibitors and other DNA-damaging agents [39, 40]. SLFN11 expression was assessed by IHC in all baseline biopsy specimens from patients receiving LMP744 at the MTD (Fig. 3, Supplementary Figure S3). SLFN11 expression was low or absent in patients 28 and 36 with colorectal cancer (nuclear SLFN11 H-scores of 0 and 32, respectively), as well as patients 31 and 33 with ovarian cancer (nuclear SLFN11 H-scores of 23 and 33, respectively), which is consistent with recent analysis of over 6,600 patient tumor biopsies [41]. Low expression in colon and ovarian tissue is consistent with published results [41, 42] and our own findings (Supplementary Figure S3). We also report low SLFN11 expression in patient 35 with ampullary cancer, a histology in which there is little known about the role of SLFN11 and drug response. In contrast, we report robust SLFN11 expression in patient 29 (SCLC, H-score of 163), consistent with the findings that higher SLFN11 expression in SCLC is associated with improved prognosis, progression-free survival, and overall survival with DNA-damaging agents [43]. The H-score of 163 is above the cut-off established by Willis et al. that distinguishes high from low SLFN11 expression (H > 122) in patients with SCLC and is associated with significantly improved PFS. Based on high SLFN11, patient 29 would have been predicted to respond to indenoisoquinoline therapy.

**Fig. 4:**
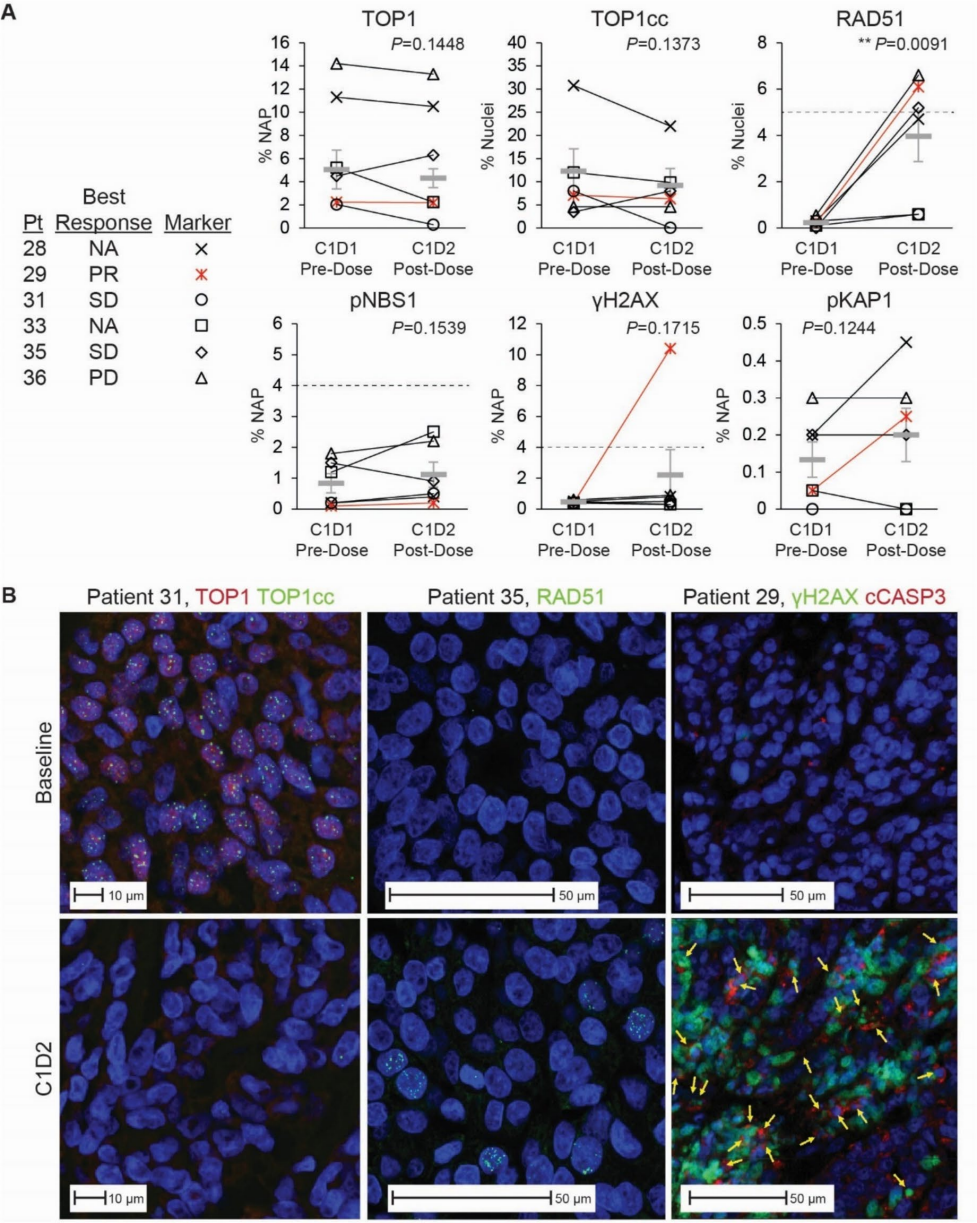
Pharmacodynamic responses to LMP744 in paired biopsies. Biomarkers of drug engagement of the TOP1 target and consequent DNA damage responses were measured in sections of pre-treatment (C1D1 pre-dose) and post-treatment (C1D2 post-dose) tumor biopsy pairs from 6 patients in the expansion cohort of LMP744 at MTD (DL6; 190 mg/m2/day), using validated quantitative immunofluorescence microscopy (qIF) assays and validated image analysis pipelines. **A** Nuclear TOP1, pNBS1, γH2AX, and pKAP1 were quantified as percent nuclear area positive (% NAP); nuclear RAD51 was quantified as % of nuclei with ≥ 5 foci; TOP1cc was quantified as the percent of nuclei with ≥ 19 foci. Intra-patient changes are shown as indicated in the legend. Mean values (± standard deviation) are shown as gray bars. In some cases, significant intra-patient changes occurred without the group mean values being significantly different (e.g., group mean values of γH2AX did not change significantly, but a γH2AX response was measured in one patient (patient 29, SCLC, cPR). **B** Representative qIF images of paired tumor biopsies with statistically significant responses in TOP1 and TOP1cc (left panels, patient 31), RAD51 (center panels, patient 35), and γH2AX (right panels, patient 29). Reflex testing of the biopsies from patient 29 for cCasp3 revealed a pharmacodynamic response to LMP744 (cells positive for both γH2AX and cCasp3) indicative of apoptosis as indicated by yellow arrows [29]

To evaluate pharmacodynamic response, we used immunofluorescence microscopy to measure the levels of TOP1 (Supplementary Figure S4), TOP1cc (Supplementary Figure S5), RAD51, pNBS1, γH2AX, and pKAP1 (Supplementary Figure S6) in paired pre-treatment and on-treatment tumor biopsies (C1D2, 2–4 h after dosing) from 6 patients receiving LMP744 at the MTD. Five of these 6 patients had received prior TOP1 inhibitor therapy (patients 28 [FOLFIRI], 29 [topotecan + berzosertib], 31 [topotecan + bevacizumab], 35 [FOLFIRINOX], and 36 [FOLFIRI + bevacizumab]). LMP744 treatment increased the level of nuclear γH2AX in tumor cells above 4% NAP (i.e., the assay threshold to exceed biological variability [15, 19, 29]) only in patient 29 with SLFN11-positive SCLC (Fig. 4A), and importantly, this γH2AX increase was accompanied by an increase in co-localized cytoplasmic cCASP3 (Fig. 4B), indicative of apoptosis [29]. Despite the γH2AX response in biopsies from patient 29, the mean values demonstrated an overall lack of treatment effect within the cohort (test of mean values *P* = 0.1715, Fig. 4A), consistent with the absence of tumor regression in the other patients in the MTD cohort. In addition, the biopsied tumor from patient 29 also exhibited pharmacodynamic responses of nuclear RAD51 (at least 5% of cells with ≥ 5 foci) and pKAP1, which indicated early responses to LMP744-induced DNA damage. Significant drug-induced increases in nuclear RAD51 were measured in 4 of 6 patients (p = 0.0091 for group effect on the mean), but nuclear pKAP1 increased only in patient 29 (Fig. 4A). LPM744 treatment failed to elicit a pNBS1 pharmacodynamic response (assay values never ≥ 4% NAP). Finally, there were no significant changes in nuclear TOP1 and TOP1cc in the biopsy cohort with LMP744 treatment (*P* = 0.1448 and *P* = 0.1373, respectively); at the individual patient level (Fig. 4B), both biomarkers decreased nearly to background levels in patient 31 (SD for 3 cycles, prior topotecan + bevacizumab treatment) and nuclear TOP1 decreased by 57% in patient 33 (unevaluable clinical response), but surprisingly, neither biomarker changed with treatment in patient 29 (Fig. 4A).

### Preclinical evaluations of LMP744 with olaparib

To investigate if SLFN11 is a dominant determinant of IIQ response in c-MYC-driven tumors that harbor fully functional BRCA1/BRCA2, which is relevant to the SCLC tumor in patient 29, the efficacy of LMP744 (10 mg/kg IV), olaparib (27 mg/kg PO), or the combination of both agents were administered to mice bearing the MYC-amplified neuroendocrine human tumor xenograft model 144,126–210-T (Supplementary Table S2), obtained from the NCI’s PDMR (https://pdmr.cancer.gov/), which is negative for nuclear SLFN11 and harbors wild-type BRCA1 and BRCA2 sequences and c-MYC amplification (> 16 gene copies), https://pdmdb.cancer.gov/web/apex/f?p=101:67). Single-agent LMP744 treatment slowed tumor growth relative to vehicle controls and single-agent olaparib treatment [40], but it did not induce tumor regression (Supplementary Figure S7A). The combination of LMP744 with olaparib resulted in additional tumor growth delay (stable disease for 3–4 weeks after the last dose) but not tumor regression. BRCA-deficiency and BRCA1-, BRCA2-, and PALB2-deficiency are genomic determinants of response to IIQs and olaparib [40, 44], respectively. As SCLC rarely harbors BRCA1/2 mutations [45], the laboratory and clinical findings point to a potentially dominant role of SLFN11 in IIQ sensitivity even in the context of MYC amplification and BRCA proficiency.

## Discussion

Following extensive preclinical development efforts, three IIQ TOP1 inhibitors have been advanced to clinical trials by the National Cancer Institute. The anti-TOP1 activity of these IIQ compounds in biochemical and cell-based assays has been evaluated extensively [11, 12, 46–48]. The safety, pharmacology, and clinical efficacy of these agents were evaluated in two phase 1 clinical trials (NCT01051635 and NCT03030417). Here, we present data for LMP776 and LMP744 and compare them to previously reported results from the phase 1 trial of indotecan (LMP400) [17]. The LMP776 study was designed to determine the MTD and evaluate pharmacodynamic endpoints. Given the lack of objective responses and short durations of stable disease, pharmacodynamic analyses could not be correlated with clinical outcomes. Accrual to the trial was closed upon MTD determination and collection of the minimum number of paired biopsies for pharmacodynamic analyses. The LMP744 study was similarly designed to determine the MTD and evaluate pharmacodynamic endpoints; accrual to this study was also stopped once enough paired biopsies had been collected from patients in the MTD expansion cohort. As the LMP744 trial was opened after completion of the LMP776 trial, newly developed, fit-for-purpose clinical pharmacodynamic assays were available for analysis of molecular tumor response to LMP744 and precluded direct comparisons of markers between the two agents.

All 3 IIQs demonstrated acceptable safety profiles at their respective MTDs [17]. The percentage of patients experiencing diarrhea (3% on each agent, all grade 2) is lower than what is reported with irinotecan treatment [49]. Given the objective of phase 1 clinical trials of establishing safety profiles and MTDs, these studies were open to patients with advanced solid tumors or lymphomas with the intention of using the preliminary efficacy data to inform patient selection in subsequent phase 2 trials if justified. The best response in patients receiving either LMP400 or LMP776 was stable disease. One patient out of 35 receiving LMP744 had a confirmed partial response (3%), who was the only 1 of 90 patients receiving any of the 3 LMP compounds on this schedule with a confirmed objective response [17]). Notably, this patient’s SCLC tumor was positive for SLFN11, which is a dominant determinant of tumor response to TOP1-targeted agents from both the IIQ and CPT classes [40]. The response rates in human subjects for the 3 IIQs were much lower than those reported in our canine lymphoma trial [16], with a best response of stable disease in patients receiving either LMP400 or LMP776. However, only 2 patients with lymphoma were accrued across trials of all 3 IIQs, a reflection of the phase 1 “all comers” study designs. The higher response rates measured in the canine trial could be due to greater activity in lymphomas. Tumor re-staging was performed after every 2 cycles (i.e., every 8 weeks) in the clinical studies versus weekly in the canine study. The short duration of responses of canine lymphoma raises the question of whether brief responses occurred in any of the patients enrolled in the IIQ trials presented here that were not captured by the evaluation schedule [16].

The 2 patients with lymphoma (Hodgkin lymphoma and cutaneous T-cell lymphoma) who enrolled in these studies both remained on study longer than the median number of cycles. One of these patients (LMP744 patient 8, Hodgkin lymphoma) achieved an unconfirmed PR before coming off study due to grade 4 leukopenia, neutropenia, and lymphopenia and grade 3 anemia, febrile neutropenia, and hypoxia; this patient was the only patient with lymphoma to receive LMP744. Notably, this patient also had the longest drug half-life measured in this study (23.4 h), raising the possibility that prolonged drug exposure may have contributed to the initial PR and/or the AEs that led to the patient’s withdrawal from the study. The patient with cutaneous T-cell lymphoma remained on study for 4 cycles while experiencing stable disease as a best response. The short durations of responses of canine lymphomas coupled with the lack of efficacy and presence of side effects in human subjects precluded further study recommendations.

The in vitro cytotoxic activities of LMP400, LMP776, and LMP744 have been correlated with expression of SLFN11 and deficient BRCA1, BRCA2, and/or PALB2 function [40]. SLFN11 prevents DNA repair via homologous recombination (HR) by irreversibly binding single-stranded DNA complexed with replication protein A (RPA) and its downregulation has also been associated with resistance to PARP inhibitors [28]. Limited preclinical combination studies of LMP744 with olaparib were evaluated in a neuroendocrine xenograft model (i.e., a histology like the tumor in patient 29 that responded to LMP744 treatment), but only an additive effect in slowing tumor growth was measured (Supplementary Figure S7). This model has low SLFN11 expression by RNA-Seq and IHC and does not harbor any known oncogenic BRCA1 or BRCA2 mutations, but it does have MYC and EGFR amplifications. This profile supports the importance of BRCA deficiency and SLFN11 expression in the synergistic effect of IIQs with olaparib [40]. Synergistic combinatorial effects have been confirmed recently for TOP1 and PARP inhibitors in glioblastoma cells, especially in PTEN-null lines [50]. There is enough interest in this combination of drug targets that efforts are underway to develop compounds capable of dual inhibition of TOP1 and PARP1 [51].

Development of non-camptothecin TOP1 inhibitors is justified because CPT and its derivative agents suffer from three major limitations: 1) rapid hydrolysis and inactivation in the blood stream and tissues at neutral pH [6, 7], 2) export of the drugs from cells by membrane pumps [8], and 3) potentially severe diarrheas in the case of irinotecan. Newer camptothecin derivatives derived from exatecan or SN38, the active metabolite of irinotecan, have recently been approved as antibody–drug conjugate (ADC) payloads by the FDA under the trade names of Enhertu (Trastuzumab Deruxtecan) and Trodelvy (Sacutuzumab Govitecan), and additional camptothecin-based ADCs are being developed [52–55]. The pharmacodynamic biomarkers derived from studies of the camptothecin TOP1 inhibitors (γH2AX, TOP1, TOP1cc, RAD51 and pNBS1) are applicable to investigations of indenoisoquinolines ([40, 54, 56] and present study). Decreased nuclear TOP1 is an established measurement of the consequence of TOP1 trapping on chromatin and subsequent proteolytic degradation [35, 57–59]. TOP1cc foci decreased in these patients’ biopsies at levels corresponding to the decreases in total TOP1. Surprisingly, we did not observe TOP1 modulation in patient 29 (PR). This profile is notably different from topoisomerase inhibitors and is consistent with reports that there is a secondary target of indenoisoquinolines [60–62].

The fluoroindenoisoquinoline LMP517, a second-generation IIQ derived from LMP744, generates both TOP1 and TOP2 cleavage complexes and has demonstrated *in vivo* efficacy against SCLC xenografts [56]. Recent IIQ derivatives have also demonstrated both stabilization of G-quadruplexes in the *MYC* promoter and inhibition of TOP1 [63]. Additional compounds have been identified based on other molecular scaffolds (i.e., not IIQs) that inhibit TOP1 and Myc while maintaining similar interactions as IIQs in molecular docking studies [64]. LMP400 recently demonstrated synergistic cell killing activity against PTEN-null glioblastoma cells when combined with the PARP inhibitor niraparib [50], results that led the FDA to grant orphan drug designation to LMP400 for use in patients with glioma. However, work on these compounds remains at preclinical stages. There are currently no active clinical trials of IIQ compounds. The finding that tumor expression of SLFN11 was high in the only patient with a cPR in the LMP744 trial, but negative in the other patients in the MTD cohort, is consistent with published studies. Higher rates of elevated SLFN11 expression have been reported in SCLC (76%) compared to other tumor histologies represented by our study population (e.g., 5% of colorectal tumors) [41]. Additional preclinical evaluations may yield evidence of potential anti-tumor activity against specific histologies, biomarker profiles, or drug combinations. Those studies will inform potential directions of future development of this class of agents.

## Supplementary Information

Below is the link to the electronic supplementary material.

Supplementary file 1 (PDF 1750 KB)

## Data Availability

De-identified clinical data will be posted on ClinicalTrials.gov (Identifiers: NCT01051635 for LMP776 and NCT03030417 for LMP744). Requests for pharmacodynamic and pharmacokinetic data may be made to the corresponding author.
