# Supplementary material for: Phase 1 studies of the indenoisoquinolines LMP776 and LMP744 in patients with solid tumors and lymphomas
Source: Cancer Chemother Pharmacol. 2025 May 29;95(1):58. doi: 10.1007/s00280-025-04778-5 (PMC12122562; doi:10.1007/s00280-025-04778-5)
Supplement: Supplementary file 1 — (PDF 1750 KB) [file 280_2025_4778_MOESM1_ESM.pdf]

Supplementary Materials

Supplementary Methods

Laboratory criteria for eligibility for LMP776 study:

Absolute neutrophil count:	≥1,500/µL
Platelets:	≥100,000/µL
Total bilirubin*:	≤1.5x Institutional upper limit of normal (ULN)
AST (SGOT)/ALT(SGPT):	≤2.5x Institutional ULN
Serum creatinine:	≤1.5x Institutional ULN
OR:	
Creatinine clearance:	≥60mL/min for patients with serum creatinine levels >1.5x higher than institutional ULN

* Patients with Gilbert's syndrome with total bilirubin up to 2.5 mg/dL were allowed.

Laboratory criteria for eligibility for LMP744 study:

Leukocytes:	≥3,000/µL
Absolute neutrophil count:	≥1,500/µL
Platelets:	≥100,000/µL
Total bilirubin:	Within normal institutional limits
AST (SGOT)/ALT(SGPT):	≤2.5x Institutional upper limit of normal (ULN)
Serum creatinine:	≤1.5x Institutional ULN
OR:	
Creatinine clearance:	≥60mL/min/1.73 m^2 for patients with serum creatinine levels >1.5x higher than institutional ULN

Supplementary Table S1: Dose levels.

	LMP776		LMP744	
Dose Level	Dose (mg/m^2)	Patients Enrolled	Dose (mg/m^2)	Patients Enrolled
-1	0.5	0	3	0
1	1	1	6	1
2	2	7	12	1
3	3	3	24	3
4	4.5	3	48	1
5	6.75	3	96	1
6	9	3	190	18
7	12	7	260	11
8	16	7		

MTD dose levels for each agent are shaded in gray.

Supplementary Table S2: Preclinical dosing

Agent	Number of mice	Doses	Schedule
Vehicle (LMP744: 1 part 20 mM HCl/10 mM Citric Acid + 1 part 5% dextrose; Olaparib: 10% DMSO in 10% HPCD in saline)	n=12	Vehicle only	QDx5, rest, QDx5
Olaparib	n=4	27 mg/kg PO	QDx5, rest, QDx5
LMP744	n=4	10 mg/kg IV	QDx5, rest, QDx5
Olaparib+LMP744	n=4	LMP744: 10 mg/kg IV<br>olaparib: 27 mg/kg PO	LMP744: QDx5, rest, QDx5<br>Olaparib: QDx5, rest, QDx5

Supplementary Table S3: Primary tumor sites.

Primary Disease Site	Total n = 91	Indotecan[¶] (LMP400) n = 21	Indimitecan (LMP776) n = 34	LMP744 n = 36
Colon	24	7	6	11
Colon/rectum	8	1	2	5
Rectum	8	4	2	2
Lung	6		2	4
Pancreas	5		3	2
Breast	3	1	2	
Skin	3	1	2[a]	
Bile duct	2			2
Liver	2		2	
Oral cavity	2	2		
Ovary	2			2
Parotid gland	2	1	1	
Sigmoid colon	2	1		1
Stomach	2		2	
Uterus	2		2	
Adenoid	1			1
Appendix	1			1
Bladder	1	1		
Bowel	1		1	
Esophagus	1		1	
Femur	1		1	
Gastroesophageal junction	1			1
Larynx	1			1
Lymph node (inguinal)	1			1[b]
Mandible	1	1		
Mesothelium	1			1
Neck	1		1	
Pelvis	1		1	
Peritoneal cavity	1		1	
Prostate	1			1
Thyroid gland	1		1	
Vagina	1	1		
Vertebral column	1		1	

[a] The two patients with primary skin tumors include one with malignant melanoma and one with cutaneous T-cell lymphoma. [b] An inguinal lymph node was designated as the primary lesion in a patient with Hodgkin lymphoma. [¶] Previously published data shown for comparison [1].

Supplementary Table S4: Grade ≥2 adverse events with LMP776 (highest grade per patient, CTCAE v4).

Adverse Event	Total (%)	Grade	DL2 * (n=7)	DL3-DL4 (n=6)	DL5-DL6 (n=6)	DL7 (n=7)	DL8 (n=7)
Anemia	17 (50)	2	5	2	3	1	
		3		1		1	4
Lymphocyte count decreased	13 (38)	2	2	1			
		3		2	1	2	3
		4					2
Platelet count decreased	6 (18)	2					1
		3				1	3
		4					1
Neutropenia	5 (15)	2			1		1
		3					2
		4					1
Hypophosphatemia	4 (12)	2	1			1	1
		3					1
Fatigue	3 (9)	2			1		2
Hypoalbuminemia	3 (9)	2	1				2
White blood cell decreased	3 (9)	2					1
		3					1
		4					1
Aspartate aminotransferase increased	2 (6)	2				1	1
Dehydration	2 (6)	2			1		1
Dysgeusia	2 (6)	2					2
Hyponatremia	2 (6)	3				1	1
Nausea	2 (6)	2					2
Alkaline phosphatase increased	1 (3)	3				1	
Diarrhea	1 (3)	2			1		
Hypercalcemia	1 (3)	4	1				
Hypoglycemia	1 (3)	3	1				
Hypokalemia	1 (3)	3					1
Hypomagnesemia	1 (3)	2					1
Hypothyroidism	1 (3)	2					1
Mucositis - oral	1 (3)	3				1	
Vomiting	1 (3)	2					1

* No grade ≥2 adverse events occurred in 1 patient at DL1. n values indicate the number of patients enrolled at each DL.

Supplementary Table S5: Grade ≥2 adverse events associated with LMP744 (highest grade per patient, CTCAE v4.03 and v5).

Adverse Event	Total (%)	Grade	DL1-DL5 (n=7)	DL6 (n=17)	DL7 (n=11)
Lymphocyte count decreased	14 (40)	2	1	1	4
		3		5	2
		4		1	
Anemia	13 (37)	2	2	4	7
Fatigue	8 (23)	2		6	2
GGT increased	6 (17)	2	1	3	
		3		1	1
Hypophosphatemia	6 (17)	2	1	4	1
Nausea	6 (17)	2		3	1
		3		1	1
Vomiting	6 (17)	2	1	4	1
Creatinine increased	5 (14)	2		3	2
Neutropenia	5 (14)	2		3	
		3		1	
		4		1	
White blood cell count decreased	5 (14)	2		3	1
		4		1	
Dehydration	4 (11)	2		1	1
		3		1	1
Weight loss	4 (11)	2		4	
Alkaline phosphatase increased	3 (9)	2	1	1	1
Alanine aminotransferase increased	2 (6)	2	1	1	
Anorexia	2 (6)	2		1	1
Aspartate aminotransferase increased	2 (6)	2		1	
		3			1
Blood bilirubin increased	2 (6)	2		1	1
Hypokalemia	2 (6)	3		1	1
Hypomagnesemia	2 (6)	2		1	1
Infections and infestations - NOS	2 (6)	2		2	
Proteinuria	2 (6)	2	1	1	
Platelet count decreased	2 (6)	2		1	1
Constipation	1 (3)	2		1	
Diarrhea	1 (3)	2		1	
Dry skin	1 (3)	2		1	

Edema - limbs	1 (3)	2	1		
Febrile Neutropenia	1 (3)	3		1	
Hyperglycemia	1 (3)	2		1	
Hypoalbuminemia	1 (3)	2		1	
Hyponatremia	1 (3)	2		1	
Hypothyroidism	1 (3)	2		1	
Hypoxia	1 (3)	3		1	
Infusion site extravasation	1 (3)	2		1	
INR increased	1 (3)	2			1
White blood cell count increased	1 (3)	2		1	
Pain - biopsy site	1 (3)	2		1	
Sudden death - NOS	1 (3)	5			1
Thrombophlebitis - superficial	1 (3)	2		1	
Urinary tract infection	1 (3)	2		1	

Supplementary Table S6: Grade ≥2 adverse events (highest grade per patient)

Adverse Event	Total (%)	LMP400[¶] (n=21) Grade 2/3/4	LMP776 (n=34) Grade 2/3/4	LMP744 (n=35) Grade 2/3/4 *
Anemia	40 (44)	8 / 2 / -	11 / 6 / -	13 / - / -
Lymphocyte count decreased	34 (38)	4 / 3 / -	3 / 8 / 2	6 / 7 / 1
Fatigue	15 (17)	3 / 1 / -	3 / - / -	8 / - / -
Neutropenia	14 (16)	1 / - / 3	2 / 2 / 1	3 / 1 / 1
White blood cell count decreased	12 (13)	1 / 1 / 2	1 / 1 / 1	4 / - / 1
Platelet count decreased	11 (12)	- / - / 3	1 / 3 / 1	3 / - / -
Hypophosphatemia	10 (11)	1 / - / -	3 / - / -	6 / - / -
Nausea	9 (10)	1 / - / -	2 / - / -	4 / 2 / -
Vomiting	7 (8)		1 / - / -	6 / - / -
Aspartate aminotransferase increased	6 (7)	2 / - / -	2 / - / -	1 / 1 / -
GGT increased	6 (7)			4 / 2 / -
Alanine aminotransferase increased	5 (6)	3 / - / -		2 / - / -
Creatinine increased	5 (6)			5 / - / -
Dehydration	5 (6)		2 / - / -	2 / 1 / -
Weight loss	5 (6)	1 / - / -		4 / - / -
Alkaline phosphatase increased	4 (4)		- / 1 / -	3 / - / -
Blood bilirubin increased	4 (4)	2 / - / -		2 / - / -
Hypoalbuminemia	4 (4)		3 / - / -	1 / - / -
Diarrhea	3 (3)	1 / - / -	1 / - / -	1 / - / -
Febrile Neutropenia	3 (3)	- / 2 / -		- / 1 / -
Hypokalemia	3 (3)		- / 1 / -	- / 2 / -
Hypomagnesemia	3 (3)		1 / - / -	2 / - / -
Hyponatremia	3 (3)		2 / - / -	1 / - / -
Anorexia	2 (2)			2 / - / -
Dysgeusia	2 (2)		2 / - / -	
Hypothyroidism	2 (2)		1 / - / -	1 / - / -
Infections and infestations - NOS	2 (2)			2 / - / -
Pain - biopsy site	2 (2)			2 / - / -
Proteinuria	2 (2)			2 / - / -
Constipation	1 (1)			1 / - / -
Dry skin	1 (1)			1 / - / -
Dyspepsia	1 (1)	1 / - / -		
Edema - limbs	1 (1)			1 / - / -
Hypercalcemia	1 (1)		1 / - / -	

Hyperglycemia	1 (1)			1 / - / -
Hyperkalemia	1 (1)	1 / - / -		
Hypoglycemia	1 (1)		- / 1 / -	
Hypoxia	1 (1)			- / 1 / -
Infusion site extravasation	1 (1)			1 / - / -
INR increased	1 (1)			1 / - / -
Lethargy	1 (1)	1 / - / -		
White blood cell count increased	1 (1)			1 / - / -
Mucositis - oral	1 (1)		- / 1 / -	
Thrombophlebitis - superficial	1 (1)			1 / - / -
Urinary tract infection	1 (1)			1 / - / -

* A patient receiving LMP744 experienced grade 5 sudden death – NOS, considered possibly related to study drug. ¶ Previously published data shown for comparison [1].

Supplementary Table S7: Non-compartmental PK parameters

LMP776

Dose Level	Dose (mg/m²)	n	C_{max} (ng/mL)	t_{max} (h)	$t_{1/2}$ (h)	AUC_{last} (ng*h/mL)	AUC_{inf} (ng*h/mL)	V_d (L/m²)	V_{ss} (L/m²)	Cl (L/h/m²)
DL1	1	1	46.9 (N/A)	1.0 (N/A)	38.6 (N/A)	359.3 (N/A)	879.6 (N/A)	63.4 (N/A)	57.9 (N/A)	2.78 (N/A)
DL2	2	7	64.4 (19.4)	1.0 (0.0)	20.8 (21.1)	530.1 (145.1)	1057.6 (1048.5)	58.4 (14.2)	54.7 (13.2)	4.0 (1.1)
DL3	3	3	124.4 (32.5)	1.0 (0.0)	13.6 (3.3)	1152.4 (211.2)	1581.5 (328.0)	37.5 (7.2)	35.4 (7.8)	2.7 (0.5)
DL4	4.5	3	178.3 (24.9)	1.0 (0.0)	13.8 (4.8)	1304.4 (366.9)	1853.2 (778.9)	49.7 (4.4)	44.0 (2.2)	3.7 (1.2)
DL5	6.75	3	273.1 (89.9)	1.0 (0.0)	11.5 (2.9)	1976.7 (746.1)	2510.3 (1078.7)	48.2 (13.6)	41.7 (11.0)	3.8 (1.6)
DL6	9	2	296.5 (64.2)	1.0 (0.0)	31.4 (21.0)	2972.1 (1315.1)	7202.3 (6047.5)	62.7 (14.7)	55.8 (8.8)	3.4 (1.5)
DL7	12	6	522.9 (161.3)	1.1 (0.2)	12.6 (2.7)	4266.7 (1392.7)	5887.2 (2205.9)	40.7 (12.2)	38.5 (10.9)	3.2 (1.5)
DL8	16	7	770.4 (262.9)	1.1 (0.4)	13.4 (5.4)	6168.1 (2341.3)	8916.3 (4523.4)	39.5 (18.8)	37.1 (15.3)	3.0 (1.4)

LMP744

Dose Level	Dose (mg/m2)	n	C_{max} (ng/mL)	t_{max} (h)	$t_{1/2}$ (h)	AUC_{last} (ng*h/mL)	AUC_{inf} (ng*h/mL)	V_d (L/m^2)	V_{ss} (L/m^2)	Cl (L/h/m^2)
DL1	6	1	48.4 (NA)	1.0 (N/A)	23.4 (N/A)	91.7 (N/A)	142.0 (N/A)	ND*	1033.0 (N/A)	42.3 (N/A)
DL2	12	1	69.6 (N/A)	1.0 (N/A)	19.1 (N/A)	116.9 (N/A)	162.3 (N/A)	ND	1369.0 (N/A)	74.0 (N/A)
DL3	24	3	78.1 (49.6)	1.6 (1.0)	14.0 (2.1)	234.2 (57.5)	312.8 (47.5)	ND	1312.7 (491.7	77.9 (11.2)
DL4	48	1	446.0 (N/A)	1.0 (N/A)	15.5 (N/A)	632.0 (N/A)	800.0 (N/A)	ND	828.0 (N/A)	72.7 (N/A)
DL5	96	1	433.1 (N/A)	1.0 (N/A)	16.2 (N/A)	1387.7 (N/A)	1857.8 (N/A)	ND	836.6 (N/A)	51.7 (N/A)
DL6	190	16	1569.0 (1078.6)	1.0 (0.1)	13.6 (7.7)	3771.8 (3247.5)	4645.2 (4158.2)	ND	845.0 (716.7)	59.1 (26.6)
DL7	260	10	1505.0 (615.0)	1.0 (0.0)	10.9 (4.1)	3583.7 (1465.3)	4096.4 (1430.4)	ND	867.8 (672.5)	73.8 (35.4)

* Not determined

Supplementary Figure S1: TOP1 function and inhibitors

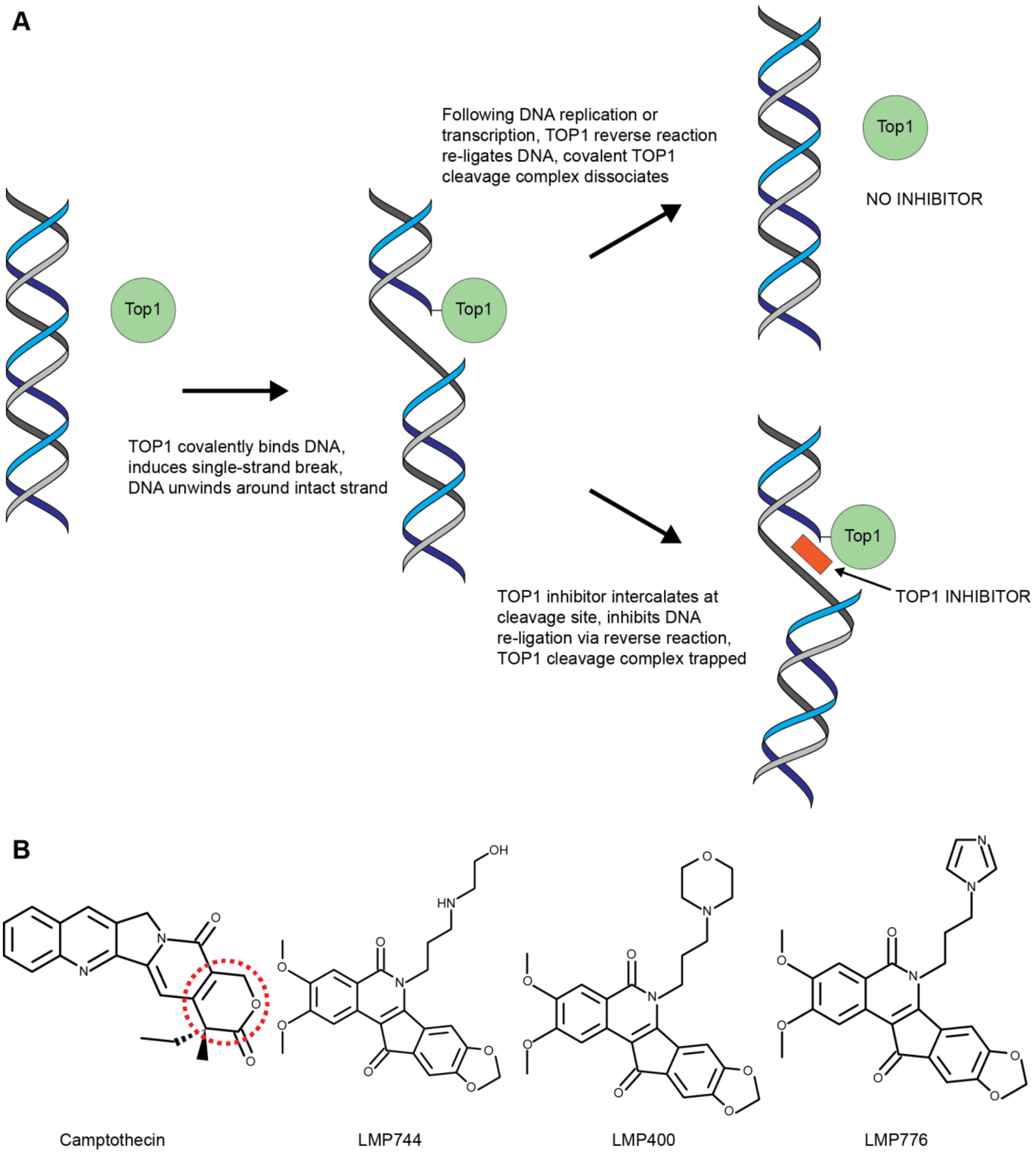

Supplementary Figure S1: Pharmacology and chemical structures of TOP1 inhibitors. (A) TOP1 inhibitors (*e.g.*, camptothecin, LMP compounds) intercalate into DNA at the TOP1

cleavage site, inhibiting the completion of the enzymatic reaction in which DNA is re-ligated and TOP1 dissociates from DNA. (B) Structures of camptothecin and the 3 LMP compounds that have completed phase 1 clinical trials. The red circle on camptothecin indicates the lactone E-ring that is susceptible to hydrolysis and consequent loss of pharmacological activity.

Supplementary Figure S2: Pharmacokinetics of LMP776 and LMP744

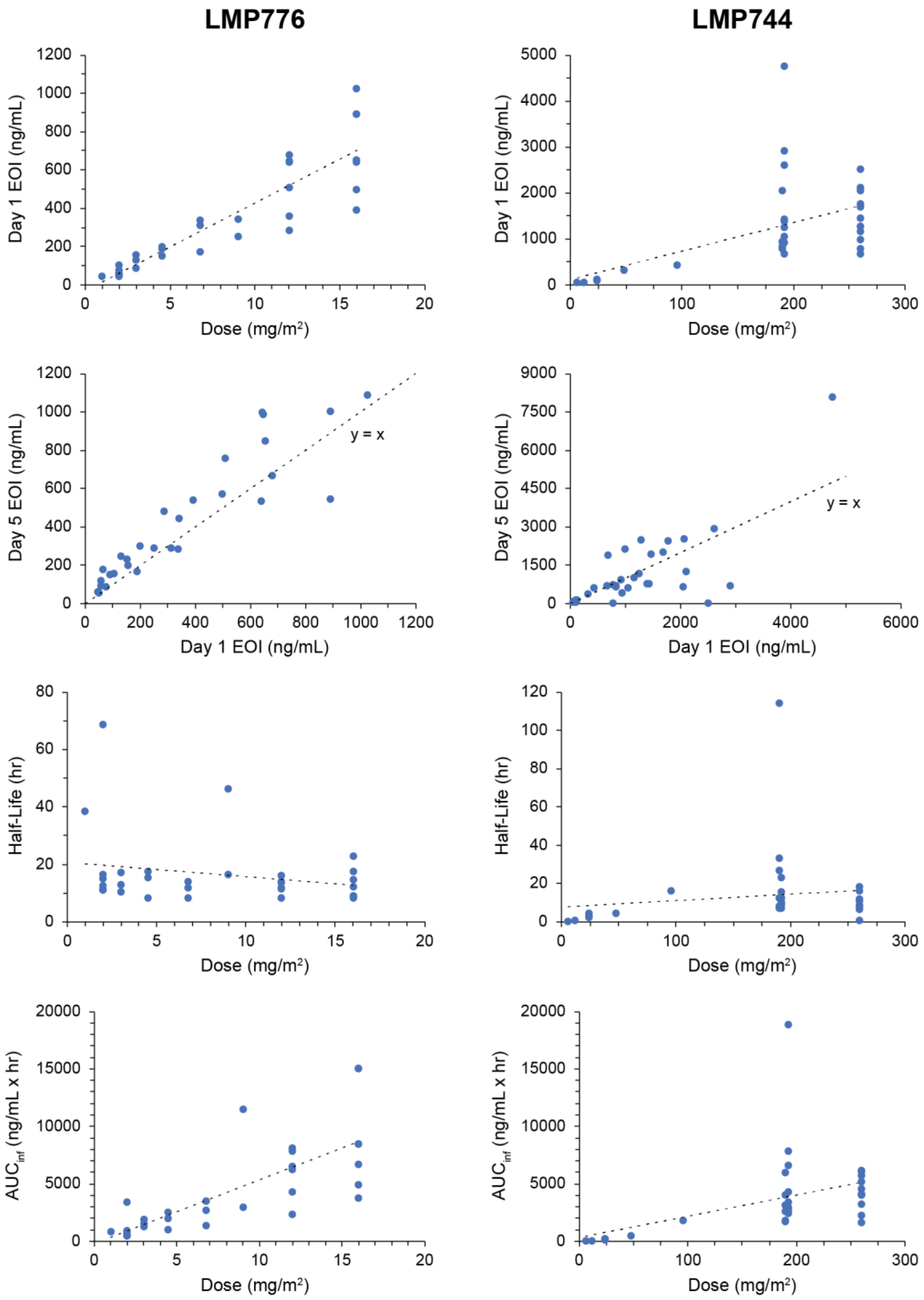

Supplementary Figure S2: Plasma pharmacokinetics of LMP776 (left, 32 patients) and LMP744 (right, 33 patients). Four DLs (1, 2, 4, and 5) included only 1 patient each. End-of-Infusion concentrations of LMP776 (left) and LMP744 (right) measured on day 1 (1st row) and day 5 (2nd row) of cycle 1 exhibited a linear relationship with dose level, and these concentrations did not increase significantly from day 1 to day 5. (3rd row) Systemic half-lives of LMP776 (left) and LMP744 (right) did not vary with dose level. (4th row) Systemic exposure (AUC) to LMP776 (left) and LMP744 (right) increased linearly with dose level. *P*-values were calculated using two-tailed paired t-tests.

Supplementary Figure S3: Validation of SLFN11 antibody specificity and performance on FFPE tissue

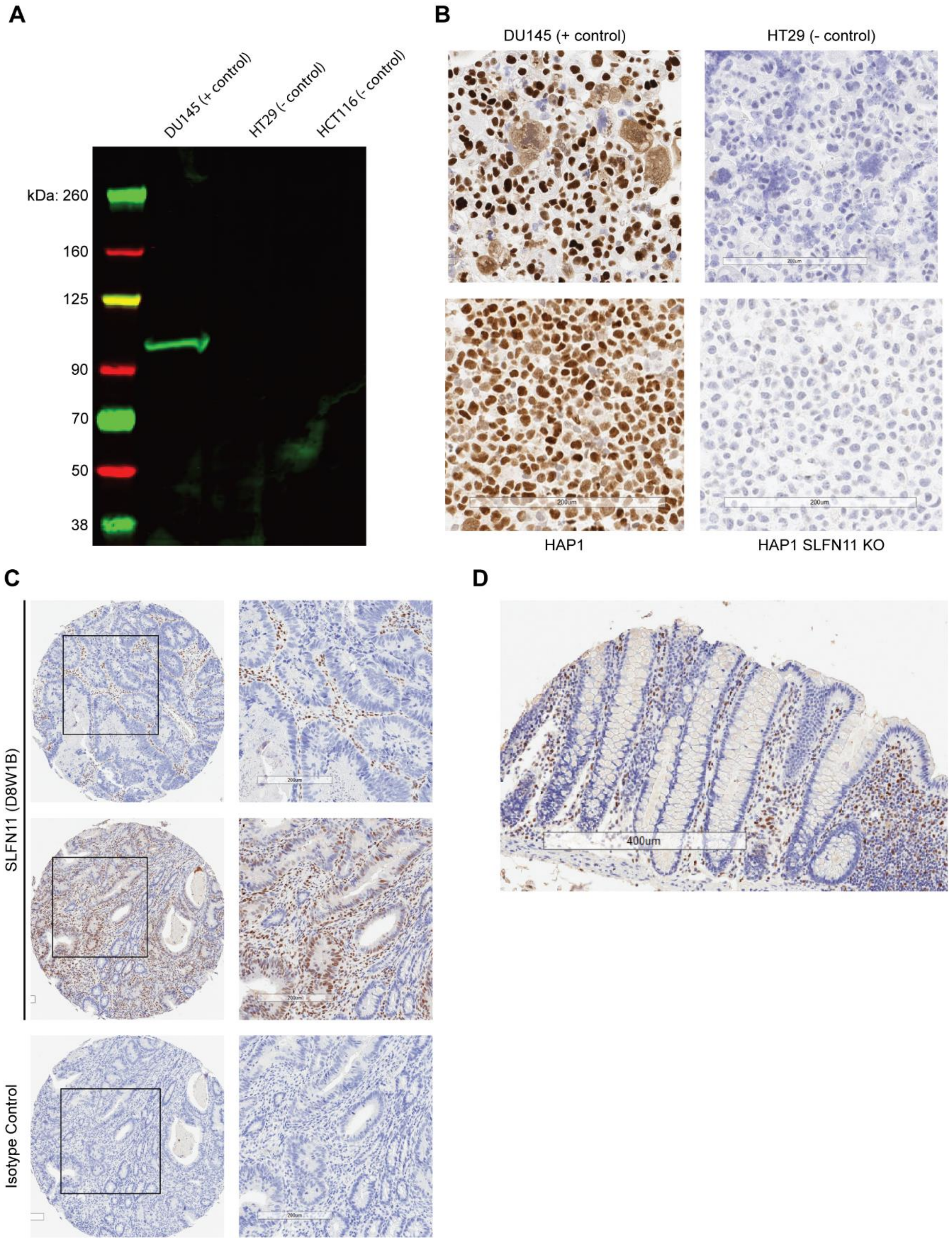

Supplementary Figure S3: Validation of SLFN11 antibody specificity and performance on FFPE tissue. A) Western blots of whole cell lysates of cell lines established as positive or negative controls for SLFN11 on the basis of mRNA levels (CellMiner [2]) probed with the D8W1B monoclonal antibody demonstrated that SLFN11 staining was only detected in cell lines that express SLFN11 mRNA. B) SLFN11 staining was only detected by IHC in FFPE in cell pellets of lines that express SLFN11 mRNA using the D8W1B monoclonal antibody and an HRP-conjugated secondary antibody. C) IHC staining for SLFN11 was performed on a section of a tissue microarray of colonic adenocarcinoma specimens obtained from Indivumed GmbH (Hamburg, Germany) using the D8W1B monoclonal antibody. Specimens were derived from resections of tumor or normal adjacent tissue and ischemia times were less than 5 minutes between tissue excision and fixation in formalin or snap freezing. Formalin fixed, paraffin-embedded (FFPE) cores were sectioned at 5 microns. The majority ($\geq$90%) of colorectal cancer specimens contained malignant cells that were SLFN11- negative, even when stromal cells exhibited strong nuclear staining (top panel). The remaining tumor cases show positive nuclear staining for SLFN11 both in the malignant CRC cells and the stromal cells (middle panel). Specificity of IHC staining of SLFN11 with monoclonal antibody clone D8W1B was further verified by the absence of staining with a rabbit IgG isotype control (bottom panel). D) SLFN11 is not expressed in FFPE sections of normal colon epithelial tissue but is expressed in the underlying stroma.

Supplementary Figure S4: Validation of TOP1 antibody specificity and performance on FFPE tissue

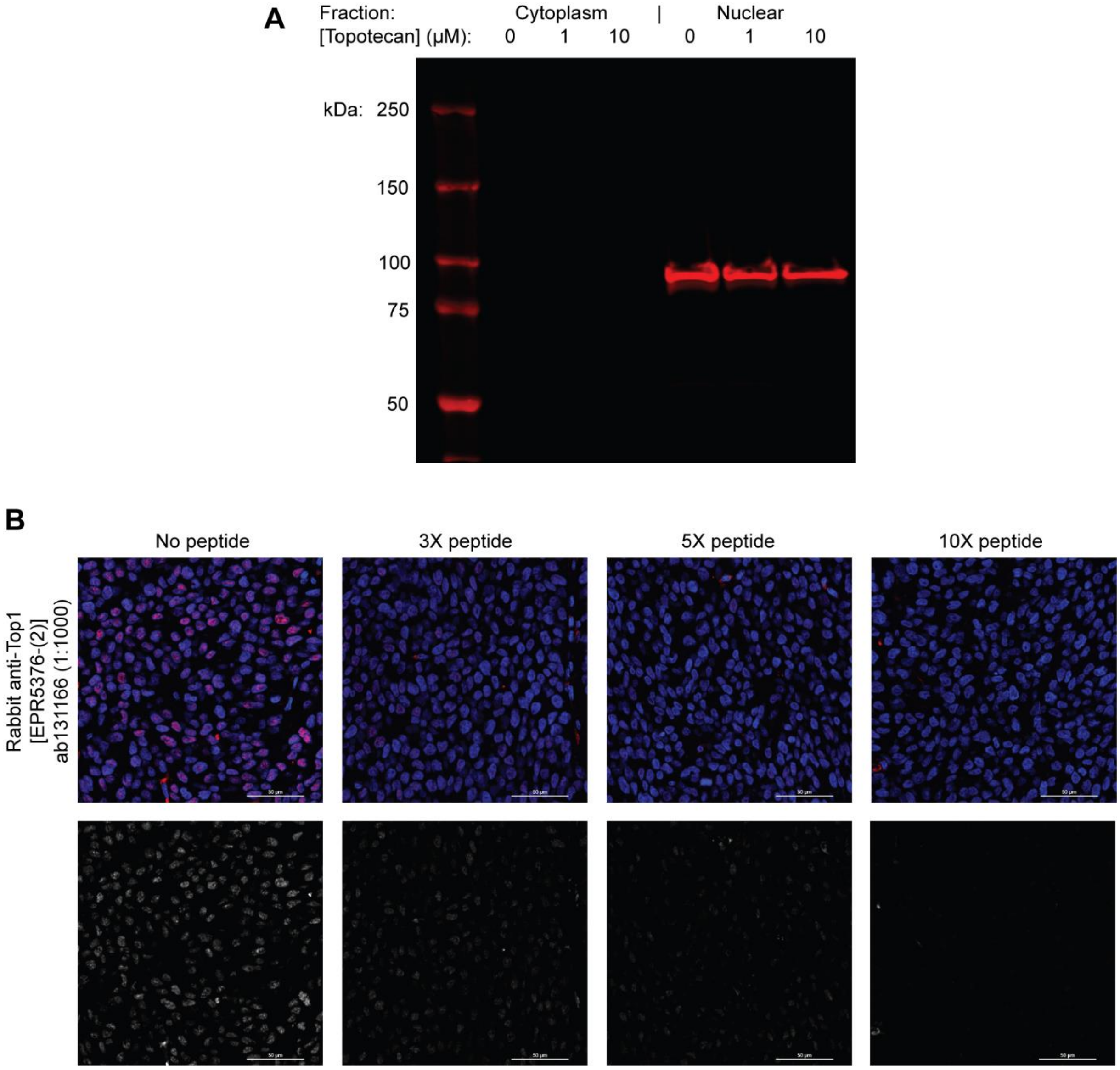

Supplementary Figure S4: Validation of TOP1 antibody specificity and performance on FFPE tissue. A) TOP1 was detected only in the nuclear fraction by Western blot of HCT116 cells treated *in vitro* with indicated concentrations of topotecan or vehicle. Anti-TOP1 rabbit mAb clone EPR5376-(2) detected the correct sized band in the nuclear but not cytosolic fraction. B) Specificity of nuclear TOP1 detection in tissue is demonstrated using peptide competition and

epitope mapping. Anti-TOP1 mAb clone EPR5376-(2) was combined with either no peptide, or 3-, 5-, or 10-fold molar excess of the peptide N-^{680}TKKVVESKKKAVQRLEEQLMKLEVQATDREENKQIA^{715}-C. The peptide was able to compete the staining of nuclear TOP1 in sections of HCT116 tumor xenografts treated with 25 mg/kg 743400 and harvested at 7h. The results demonstrate that the mAb clone EPR5376-(2) specifically detected nuclear TOP1. The bottom row shows monolayer images of TOP1 without DAPI.

Supplementary Figure S5: Validation of TOP1cc antibody specificity and performance on FFPE tissue

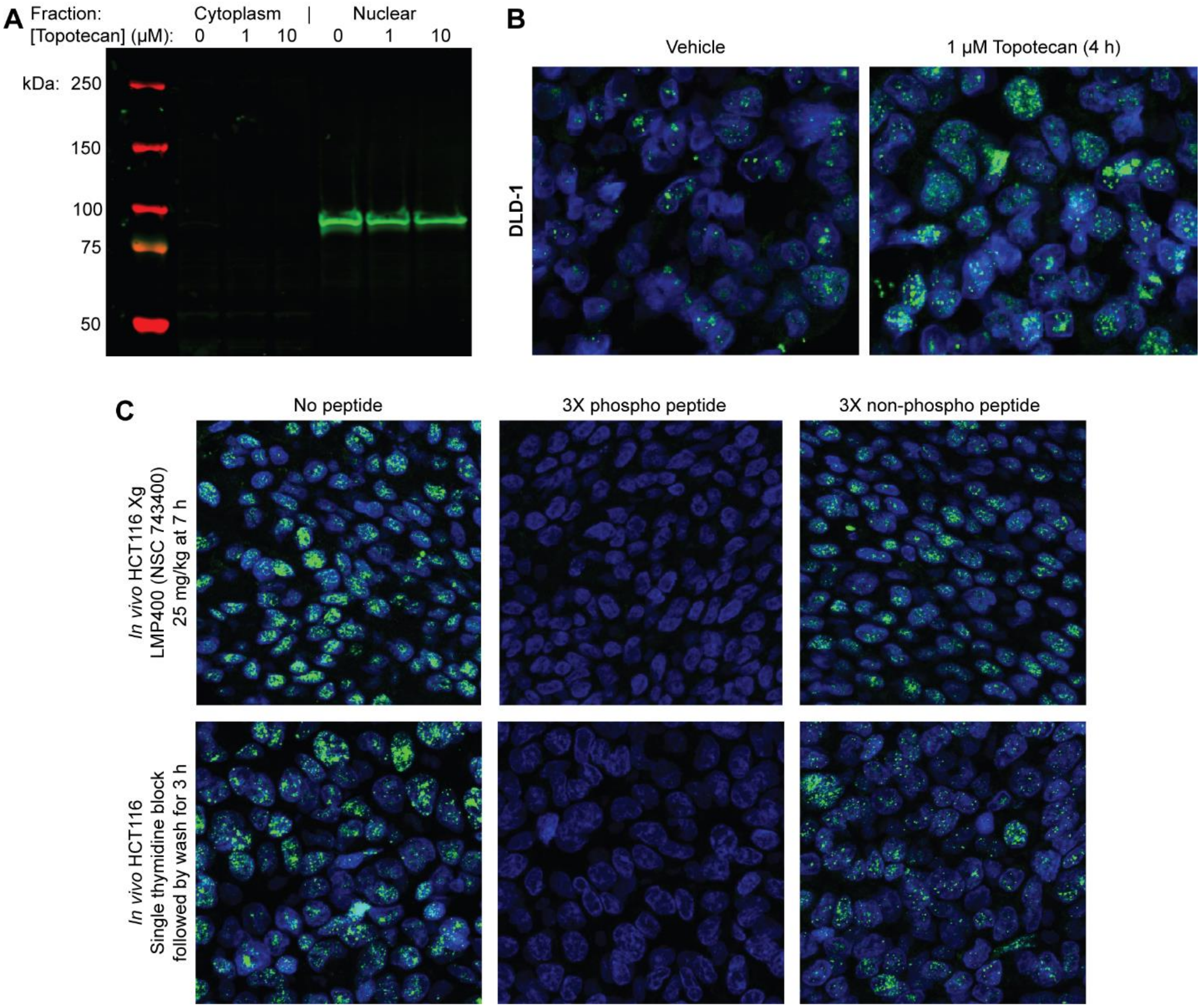

Supplementary Figure S4: Validation of TOP1cc antibody specificity and performance on FFPE tissue. A) TOP1cc's were detected only in the nuclear fraction by Western blot of HCT116 cells treated *in vitro* with indicated concentrations of topotecan or vehicle with anti-TOP1cc mAb clone 1.1A. B) The DLD1 cell line was treated with 1 uM topotecan or vehicle for 4 hours then processed into FFPE cell pellets. TOP1cc nuclear foci induction was evaluated by immunofluorescence staining and confocal imaging. C) TOP1cc mAb clone 1.1A was combined with either no peptide, 3-fold molar ratio of the phospho-specific peptide

LGTSKLN(pY)LDPRITV which is the antigen that was used to generate the antibody [3] or the unphosphorylated version of the same peptide LGTSKLNYLDPRITV. Only the phosphorylated peptide was able to compete the TOP1cc nuclear foci from samples (either HCT116 xenograft tumor treated with 25 mg/kg 743400 and harvested at 7h, or HCT116 cells grown in vitro, treated with a thymidine block, washed and harvested after 3 hours to capture cells in S-phase). The results demonstrate that the mAb clone 1.1A specifically detected TOP1cc covalent complexes.

Supplementary Figure S6: Validation of pKAP1 antibody specificity and performance on FFPE tissue

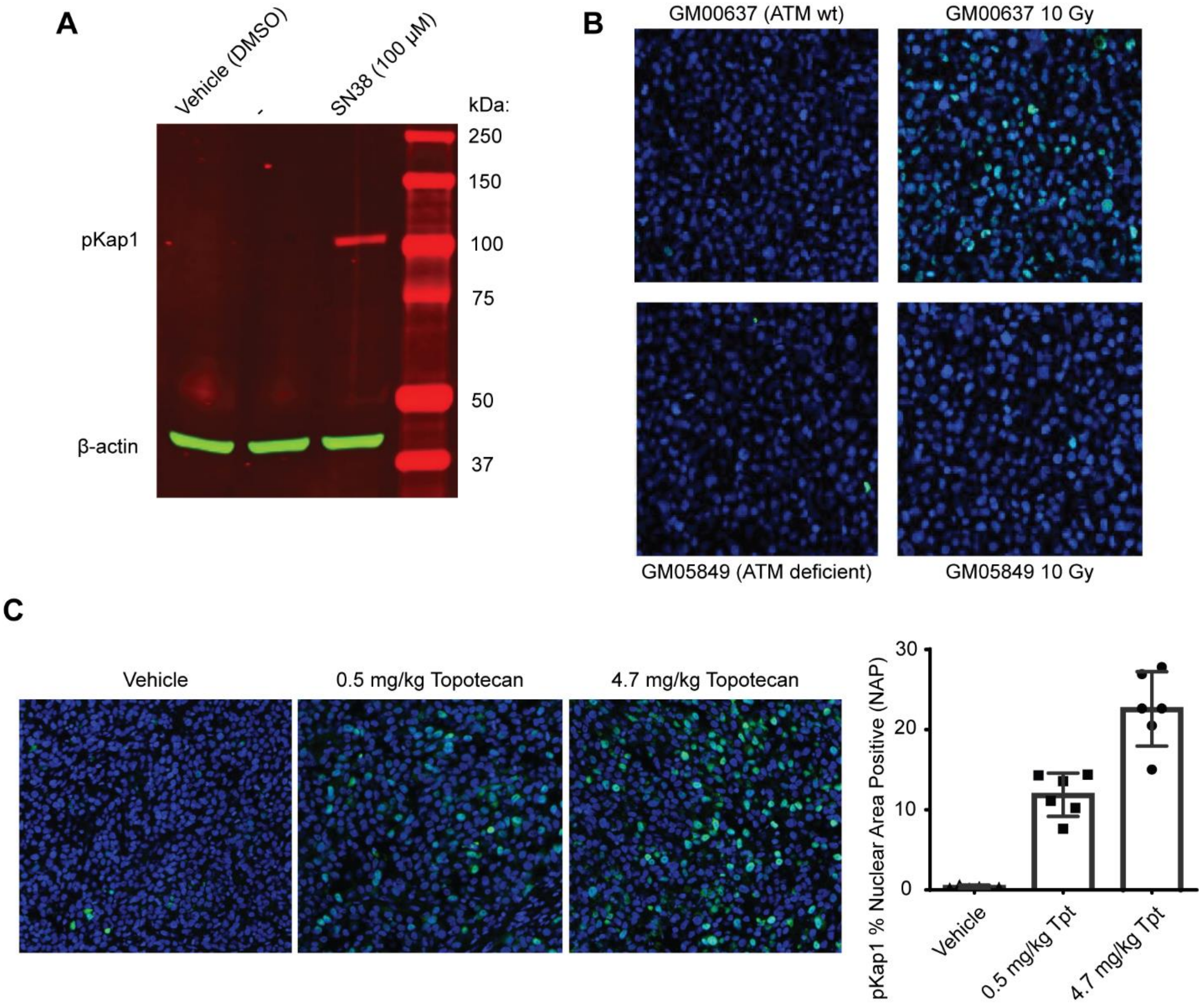

Supplementary Figure S5: Validation of pKAP1 antibody specificity and performance on FFPE tissue. A) HT29 cells were treated for 3 hours *in vitro* with SN38 and lysates were evaluated by Western blot with pKap1 (clone EPR5248). B-actin antibody in green as loading control. B) ATM-wt (GM00637) or ATM-deficient (GM05849) fibroblast cells were fixed 1 hour after receiving no treatment (left panels) or 10 Gy ionizing radiation (right) and examined for pKAP1 induction. Induced pKAP1 was detected only in the irradiated ATM wild-type fibroblast line.

C) A375 xenograft tumors treated with vehicle, 0.5 mg/kg (minimum biologically active dose), or 4.7 mg/kg (maximum tolerated dose) topotecan and harvested after 4 hours. Frozen xenograft tissue was flash frozen, then formalin-fixed, paraffin-embedded, and 5-micron sections were stained with pKAP1 and imaged on Aperio FL. Quantitative analyses of one tumor tissue quadrant from each timepoint are shown; each point on the graph represents one image field. Over 6000 nuclei total were measured per xenograft using Definiens image analysis software. The results demonstrate that the mAb clone EPR5248 specifically detected nuclear pKAP1.

Supplementary Figure S7: Tumor response of the neuroendocrine xenograft model to LMP744, olaparib, or the combination

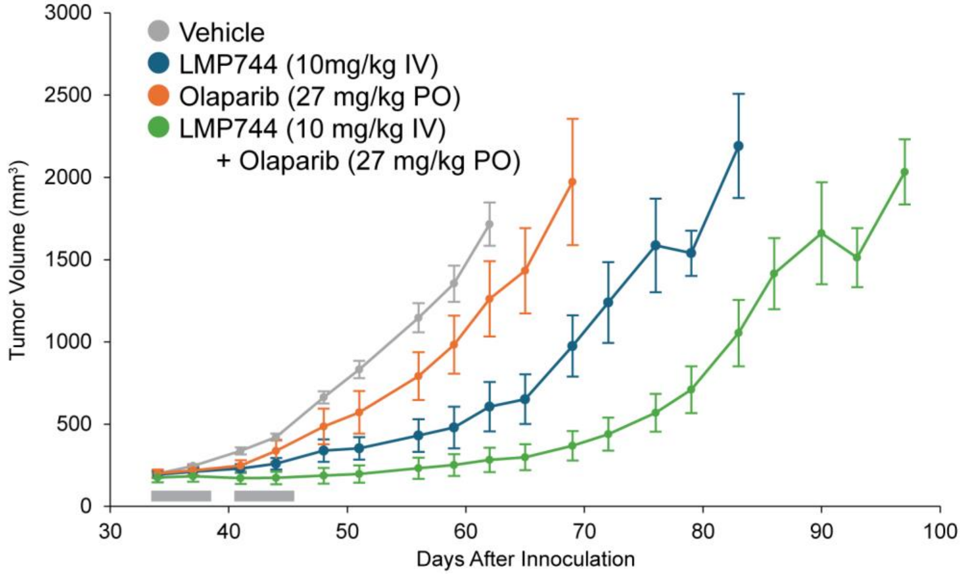

Supplementary Figure S7: Tumor response of the neuroendocrine xenograft model to LMP744, olaparib, or the combination. LMP744 and olaparib were administered as either single agents or in combination to mice bearing 144126-210-T neuroendocrine tumor patient-derived xenografts. While tumor growth was slower in mice treated with LMP744 or the combination of LMP744 with olaparib, no tumor regression occurred in any treatment arm. Graphs show tumor volume where >50% of the animals remained on study. Gray bars below tumor growth curves indicate treatment days.

Supplementary References

1. Kummar S, Chen A, Gutierrez M, Pfister TD, Wang L, Redon C, Bonner WM, Yutzy W, Zhang Y, Kinders RJ, Ji J, Allen D, Covey JM, Eiseman JL, Holleran JL, Beumer JH, Rubinstein L, Collins J, Tomaszewski J, Parchment R, Pommier Y,Doroshow JH (2016) Clinical and pharmacologic evaluation of two dosing schedules of indotecan (LMP400), a novel indenoisoquinoline, in patients with advanced solid tumors. Cancer Chemother Pharmacol 78(1):73-81. 10.1007/s00280-016-2998-6
2. Murai J, Feng Y, Yu GK, Ru Y, Tang SW, Shen Y,Pommier Y (2016) Resistance to PARP inhibitors by SLFN11 inactivation can be overcome by ATR inhibition. Oncotarget 7(47):76534-76550. 10.18632/oncotarget.12266
3. Patel AG, Flatten KS, Peterson KL, Beito TG, Schneider PA, Perkins AL, Harki DA,Kaufmann SH (2016) Immunodetection of human topoisomerase I-DNA covalent complexes. Nucleic Acids Res 44(6):2816-2826. 10.1093/nar/gkw109
